# Polymorphisms of −174G>C and −572G>C in the Interleukin 6 (IL-6) Gene and Coronary Heart Disease Risk: A Meta-Analysis of 27 Research Studies

**DOI:** 10.1371/journal.pone.0034839

**Published:** 2012-04-11

**Authors:** Guo-hua Zheng, Hai-ying Chen, Shang-Quan Xiong

**Affiliations:** 1 The Centre of Evidence Based Medicine, Academy of Integrative Medicine, Fujian University of Traditional Chinese Medicine, Shangjie University Town, Fuzhou, China; 2 The People's Hospital of Fujian Province, Fuzhou, China; Universite de Montreal, Canada

## Abstract

**Objective:**

Elevated serum IL-6 level is a risk factor for coronary heart disease (CHD). The −174G>C and −572G>C polymorphisms in the IL-6 gene have previously been shown to modulate IL-6 levels. But the association between the −174G>C and −572G>C polymorphisms and the risk of CHD is still unclear. A meta-analysis of all eligible studies was carried out to clarify the role of IL-6 gene polymorphisms in CHD.

**Methods and Results:**

PubMed, EMBASE, Vip, CNKI and CBM-disc were searched for eligible articles in English and Chinese that were published before October 2010. 27 studies involving 11580 patients with CHD and 17103 controls were included. A meta-analysis was performed for the included articles using the RevMan 5.0 and Stata 10.0 softwares. Overall, the −174C allele was not significantly associated with CHD risk (ORs = 1.04, 95%CI = 0.98 to 1.10) when compared with the −174G allele in the additive model, and meta-analysis under other genetic models (dominant, recessive, CC versus GG, and GC versus GG) also did not reveal any significant association. On the contrary, the −572C allele was associated with a decreased risk of CHD when compared with the −572G allele (ORs = 0.79, 95%CI = 0.68 to 0.93). Furthermore, analyses under the recessive model (ORs = 0.69, 95% = 0.59 to 0.80) and the allele contrast model (genotype of CC versus GG, ORs = 0.49, 95% = 0.35 to 0.70) yielded similar results. However, statistical significance was not found when the meta-analysis was restricted to studies focusing on European populations, studies with large sample size, and cohort studies by using subgroup analysis.

**Conclusions:**

The −174G>C polymorphism in the IL-6 gene is not significantly associated with increased risks of CHD. However, The −572G>C polymorphism may contribute to CHD development. Future investigations with better study design and large number of subjects are needed.

## Introduction

Coronary heart disease (CHD), including myocardial infarction (MI), angina pectoris, and aterios sclerosis of the coronary arteries, is still one of the leading causes of death and disability. CHD also accounts for a huge consumption of health care resources especially in rapidly ageing societies [1]. Though CHD's etiology seems to be multi-factorial and complicated, the most basic pathogenic factor is the atherosclerosis of coronary artery. Atherosclerosis is a chronic inflammatory response associated with several pathophysiological reactions in the vascular wall [2]–[3]. Inflammation plays a central role in the development of atherosclerosis, and is considered to be a primary risk factor [4]. The inflammatory cytokines that are present in the arterial lesions can mediate the various processes of plaque formation, plaque progression and vessel thrombosis [5]. Furthermore, plasma levels of inflammatory cytokines are also associated with atherosclerotic risk in a variety of clinical setting [6]–[8]. Interleukin-6 (IL-6) is an important pleiotropic cytokine that is produced by different cells including adipocytes, endothelial cells, fibroblasts, myocytes, and white blood cells [9]. IL-6 has a broad range of humoral and cellular immune properties relating to inflammation, and is one of the most important mediators of the inflammatory response [10]–[11]. Some studies have reported that IL-6 can influence a variety of cellular functions, such as B-cell immunoglobulin production, T-cell cytotoxic activity, platelet reactivity, vascular smooth muscle proliferation and endothelial cell activation [12]–[13]. Moreover, IL-6 can activate the endothelium facilitating leukocytes migrate into the vessel wall, and stimulate vascular smooth muscle cell proliferation [14]–[15]. In addition, IL-6 regulates production of adhesion molecules and induces secretion of monocyte chemotactic protein, an important mediator for releasing other cytokines, such as tumor necrosis factor-α and IL-1β, which subsequently enhance the inflammatory reaction [12], [15]. Epidemiological data have demonstrated that IL-6 is associated with clinical and subclinical cardiovascular diseases [16]. Recent studies have also revealed the association between IL-6 plasma levels and cardiovascular pathology [17]–[19]. Correlations have been found between elevated circulating levels of IL-6 and an increased risk of myocardial infarct in apparently healthy men, supporting a role of IL 6 in the early stages of atherogenesis [20].

The circulating concentrations of IL-6 are likely to be influenced by several environmental and genetic factors, subsequently affecting an individual's susceptibility to disease [21]–[22]. Circulating levels of inflammatory markers have been linked to an increased risk of atherosclerotic events [23]. Previous studies have also shown the association between single nucleotide polymorphisms (SNPs) in the IL-6 gene and the plasma levels of IL-6 [24]–[26]. Polymorphisms in the promoter region of the IL-6 gene may result in inter-individual variation in transcription and expression of the IL-6 gene [27]. Those SNPs of the IL-6 gene have been reported to be functionally important since they influence IL-6 transcription rates [22].

Several polymorphisms in the IL-6 promoter have been observed in healthy and unhealthy populations. Two single-nucleotide polymorphisms, the common −174 G>C variant [28] and the less frequent −572G>C allele [27], have been studied in detail. Fishman's study showed that the −174 G>C and −572 G>C polymorphisms in the IL-6 gene could increase the plasma concentrations of IL-6 [21]. Other studies have reported that the −174C allele was associated with higher plasma concentrations of IL-6 [29]–[31]. It was also suggested that the polymorphisms can influence the transcription and plasma levels of IL-6. However, other studies did not find the associations between IL-6 levels and the promoter polymorphisms [32]–[33]. It was reported that the −174 G>C polymorphism was not associated with cardiovascular death or a new myocardial infarction, whereas the −572 G>C polymorphism was associated with a borderline significant increase in risk (*P* = 0.05) in univariate analysis [34].

In summary, previous researches investigating the relationship between the −174G>C or −572G>C polymorphisms and CHD risk in different study populations have yielded varied results. In order to clarify the role of IL-6 gene polymorphisms in CHD, we have conducted a formal meta-analysis for all eligible studies published before October 2010.

## Methods

### Identification and eligibility of relevant studies

Computer-based searches of PubMed, EMBASE, and China National Knowledge Infrastructure (CNKI), VIP database for Chinese Technical Periodicals (VIP), China Biological Medicine Database (CBM-disc) were performed without restricting language while using key words relating to CHD disease (e.g., CHD OR CAD OR CVD OR “coronary disease” OR “coronary heart disease” OR “coronary artery disease” OR “ischemic heart disease” OR “cardiovascular disease” OR “heart disease”) in combination with words related to IL-6 (e.g., “interleukin 6” or IL-6 or “IL 6” OR “interleukin-6” ) and polymorphism (e.g., polymorphism OR mutation OR genetic OR genotype OR “single nucleotide polymorphism”). The searches included all articles published before October 2010. In addition, the reference lists in all relevant studies and review articles were also examined.

Only studies that evaluated the association between CHD and the IL-6 gene polymorphisms were included. Included studies also met the following criteria: (1) They were original studies containing independent data, (2) the study design was case-control study or cohort study, (3) the diagnosis of disease was definite (Relevant clinical outcome in cases should be confirmed by WHO criteria or coronary stenosis, which is defined variously as minimally 50% or 70% stenosis of at least one major coronary arteries ), (4) the −174G>C or −572G>C genotype frequencies of the IL6 gene were provided, (5) the risk of CHD was evaluated, and the odds ratio (OR) value could be calculated. The major reasons for exclusion were (1) they were family studies, (2) insufficient data about CHD, (3) lack of accessibility to original articles and duplicate publications.

### Data extraction

Two investigators independently reviewed all studies and extracted the data by using a standardized form. The following information was independently summarised from each included study: the first author, publication date, age, gender ratio, origin of country, source of controls, study design, ethnicity, definition of disease in cases, numbers of cases and controls, genotyping method, frequency and numbers for each genotype. Two reviewers compared the results from all included studies for accuracy and discussed any discrepancies before reaching to an agreement. If there were still some studies that the two reviewers failed to agree on, a third reviewer was then introduced to assess the studies.

### Statistical analysis

Deviation from Hard-Weinberg Equilibrium (HWE) was measured by χ^2^-test for the control groups of each study. If the control groups were not in HWE, sensitivity analysis or subgroup analysis was performed to test the robustness of the findings.

Meta-analysis was performed to investigate the association between the −174G>C and −572G>C polymorphisms in the IL-6 gene and the risk of CHD. We systematically assessed the risk of different genotypes of the −174G>C and −572G>C polymorphisms in CHD under various genetic models, which included the additive (allele G versus allele C), the recessive (CC versus GG+GC), the dominant (GG+GC versus CC), the allele contrast (CC versus GG) and (GC versus GG) models. The strength of association between the −174G>C or −572G>C polymorphisms and CHD risk was measured by overall odds ratios (ORs) with 95% confidence interval (95%CI).

The homogeneity between studies was assessed using Q statistic test [35] and Higgins *I* ^2^ [36]. When heterogeneity was not significant (*P*_Heterogeneity_≥0.1), the results were pooled using a fixed effect model and the Mantel-Haenszel method. Otherwise, a random-effect model and the Dersimonian and Laird method [37] were applied. Funnel plots and Begg's test were used to examine the publication bias for reported associations [38]. In addition, study design (cohort studies and case control studies), source of control (population based and hospital based), ethnicity (European, Asian, and others), sample size (>800, 400 to 800, and <400), status of Hardy-Weinberg Equilibrium (meeting or deviating) and types of CHD end points (myocardial infarction, coronary stenosis, mixed MI and CHD) were used as characteristics for assessment of heterogeneity. Furthermore, subsidiary analyses including subgroup analyses, random-effects meta-regression, and adjusted meta-analysis were performed according to above characteristics.

All analyses were performed by using the Review-manager 5.0.1 (Oxford, England) and the Stata version 10.0 (Stata Corporation, College Station, Texas. USA) softwares. All *P*-values were two-sided.

## Results

### Characteristics of the Included Studies

**Figure 1:**
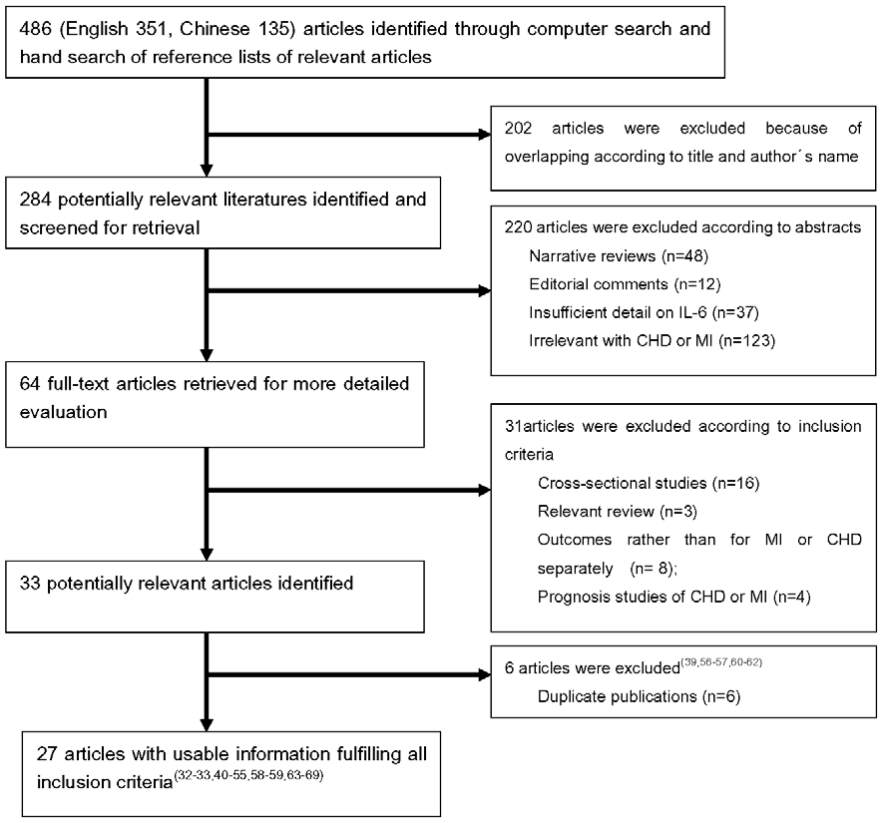
Flow diagram summarizing the search strategy for meta-analysis of IL-6 gene −174 G>C,−572G>C and CHD.

A total of 486 articles were found based on the above searching criteria. 202 overlapping articles were excluded after comparing the title and author name. 220 articles were further excluded including reviews, editorials, and studies with insufficient detail on IL-6 or irrelevant outcome with CHD or MI by reading abstracts. Detailed evaluation on the full texts of the remaining 64 articles subsequently excluded 31 articles that failed to meet the inclusion criteria. Further assessment excluded 6 ineligible articles because of duplication [39], [56]–[57], [60]–[62]. Finally, 27 articles [32]–[33], [40]–[55], [58]–[59], [63]–[69] were included in the meta-analysis (Figure 1).

### Summary of statistics

In the 27 included articles, a total of 11580 patients and 17103 controls were investigated. The characteristics of those studies in the meta-analysis were listed in Table S1 and Table S2. There were 14 studies from European countries, 11 studies from Asian countries (8 in east Asian, 3 in south Asian), 1 study from America, and 1 study from Brazil. The studies were case control in design, except for two studies that were cohort studies (Table S1). Only four studies on the −174G>C polymorphism and no study on the −572 G>C polymorphism reported the sex-specific polymorphism distribution for the IL-6 gene. The diagnoses in the included articles were CHD, CVD, MI, AMI and CAD. All of them were classified using WHO criteria or coronary arteriography (≥50% stenosis in at least one major artery). 24 studies used population-based controls, and 3 studies used hospital-based controls (Table S2). All genotypes and allele frequencies of the −174G>C and −572G>C polymorphisms in cases and controls were shown in Tables S3, S4, respectively. For the −174G>C polymorphism, 22 studies with 28 comparisons were available, including a total of 11060 cases and 16752 controls (Table S3). For the −572G>C polymorphism, 13 studies covered a total of 3255 cases and 6243 controls (Table S4). In most of the studies the polymorphisms were found to occur in frequencies consistent with HWE. However, statistically significant deviations from HWE were found in four studies (two for the −174G>C polymorphism and two for the −572G>C polymorphism), and HWE could not be calculated in two other studies (Table S3, S4).

### Association of the IL-6 gene −174G>C polymorphism with CHD risk

The association between the −174G>C polymorphism and CHD risk was investigated under the additive model (allele C versus allele G). Substantial heterogeneity among the studies (*I* ^2^ = 35%, *P* = 0.036) was found. The overall OR under a random-effects model was 1.04 (95% CI = 0.98 to 1.10), and was not significant (Figure S1). The result was stable when using the cumulate Meta-analysis according to the publication years (Figure S2). An overall analysis under all other genetic models was then performed. The results of meta-analysis did not also show a significant association under the dominant model (OR = 1.05, 95%CI = 0.95 to 1.16) and the recessive model (OR = 1.03, 95%CI = 0.95 to 1.11). Furthermore, significant association was not found under other pair-wise comparisons (allele CC versus GG, and allele GC versus GG). The results were shown in Table 1.

**Table 1:** ORs and 95% CI for coronary heart disease and the −174G>C, −572 G>C polymorphism in IL-6 gene under various genetic models.

			*I^2^*	p-value for	Statistic	Effect estimate
Genetic model	Comparisons	Participant	(%)	heterogeneity	model	OR (95% CI)
−174 G>C polymorphism						
Additive	28	53980	35	0.036	Random	1.04 (0.98, 1.10)
Recessive	28	27454	0	0.56	Fixed	1.03 (0.95, 1.11)
Dominant	28	26526	44	0.007	Random	1.05 (0.95, 1.16)
CC vs GG	28	14243	17	0.23	Fixed	1.05 (0.96, 1.15)
GC vs GG	28	21359	57	0.0002	Random	1.06 (0.94, 1.19)
−572 G>C polymorphism						
Additive	13	18442	54.2	0.01	Random	0.79 (0.68, 0.93)
Recessive	13	9221	15.1	0.29	Fixed	0.69 (0.59, 0.80)
Dominant	13	9221	49.4	0.02	Random	1.29 (0.98, 1.69)
CC vs GG	13	7498	0	0.50	Fixed	0.49 (0.35, 0.70)
GC vs GG	13	7681	39	0.07	Random	0.86 (0.67, 1.11)

In order to analyze characteristic-homogeneous groups, subgroup analyses were carried out according to study characteristics including ethnicity, type of study, sample size, source of controls and HWE status. No significant association was found under the additive genetic model by using the study characteristics mentioned above. The sensitivity analysis between the fixed model and the random model also indicated that the changes of most results were slight (Table 2).

**Table 2:** Studies of the −174G>C polymorphism in IL-6 gene and risk of coronary heart disease under additive model grouped by study characteristics.

Study			*I^2^*	Effect estimate	Effect estimate
characteristics	Comparisons	Participants	(%)	OR (95% CI) (fixed)	OR (95% CI) (random)
Over all	28	26526	35	1.03 (0.99, 1.07)	1.04 (0.98, 1.10)
Ethnicity					
European	19	22128	49	1.03 (0.98, 1.08)	1.05 [0.97, 1.12]
Asian	6	2775	0	0.98 (0.85, 1.14)	0.99 [0.85, 1.14]
Other	3	1623	28	1.11 (0.95, 1.29)	1.08 [0.89, 1.32]
Type of study					
Case control	25	18122	41	1.02 (0.97, 1.07)	1.03 [0.96, 1.11]
Cohort	3	8404	0	1.08 (0.97, 1.20)	1.08 [0.97, 1.20]
Sample size					
Large (>800)	11	21025	2.3	1.03 (0.98, 1.08)	1.16 (0.93, 1.45)
Middle(400–800)	5	2658	24.9	0.94 (0.83, 1.06)	0.94 (0.82, 1.09)
Little(<400)	12	2843	43.9	1.20 (1.04, 1.38)	1.16 (0.93, 1.45)
Control source					
Hospital	4	1117	73	1.17 (0.96, 1.42)	1.16 [0.79, 1.71]
Population	24	25409	21	1.02 (0.98, 1.07)	1.03 [0.98, 1.08]
HWE status					
Meeting HWE	24	24326	17.5	1.01 (0.97, 1.06)	1.04 (0.97, 1.07)
Deviating HWE	4	2200	66.5	1.20 (1.04, 1.39)	1.23 (0.81, 1.68)

### Association of the IL-6 gene −572G>C polymorphism with CHD risk

13 studies were included in the meta-analysis of the −572G>C polymorphism with CHD risk. Analysis of −572G>C polymorphism in the IL-6 gene with CHD risk under the additive model was performed and the random model was used to assess the overall OR value. Compared with the carrier of the G allele, the overall OR of the C allele with CHD risk was 0.79 (95%CI = 0.68 to 0.93) (Table 1, Figure S3). Under the recessive and the dominant models, the overall OR was 0.69 (95%CI = 0.59 to 0.80) and 1.29 (95%CI = 0.98 to 1.69), respectively. When pair-wise comparisons were made, comparing with the GG genotype, the overall OR of the CC genotype with CHD risk was 0.49 (95%CI = 0.35 to 0.70). However, compared with homozygotes of the G allele, significant association of the GC genotype with CHD risk was not found and the overall OR was 0.86 (95%CI = 0.67 to 1.11). Analyses under other genetic models were shown in Table 1.

According to study characteristics, the subgroup analysis and sensitivity analysis were performed under the additive model. The results showed that the −572C allele in European population had no significant effect on the risk of CHD (OR = 1.02; 95%CI:0.85–1.22), whereas this effect was reversed in Asian population (OR = 0.71; 95%CI:0.63–0.81). Subgroup analysis by sample size suggested heterogeneous associations of the −572G>C polymorphism with CHD for the −572C allele in studies with large sample sizes (OR = 1.02, 95%CI:0.85–1.22), and a protective effect in studies with intermediate or smaller sample size (OR = 0.71, 0.72; 95%CI: 0.58–0.87, 0.61–0.84). When stratifying by HWE, results did not change when restricted to the studies with the confirmed HWE. A sensitivity analysis comparing the fixed and the random models showed only minor changes in the estimates (Table 3).

**Table 3:** Studies of the −572 G>C polymorphism in IL-6 gene and risk of coronary heart disease under additive model grouped by study characteristics.

Study			*I^2^*	Effect estimate	Effect estimate
characteristics	comparisons	Participants	(%)	OR (95% CI) (fixed)	OR (95% CI) (random)
Over all	13	18442	54.2	0.80 (0.72, 0.89)	0.79 (0.68, 0.93)
Ethnicity					
European	4	12998	0	1.02 (0.85, 1.22)	1.02 (0.85, 1.22)
Asian	9	5444	54.2	0.71 (0.63, 0.81)	0.70 (0.59, 0.83)
Type of Study					
Case control	12	13218	51.5	0.79 (0.71, 0.87)	0.77 (0.66, 0.90)
Cohort	1	5224		1.26 (0.78, 2.05)	1.26 (0.78, 2.05)
Sample size					
Large (>800)	4	12998	0	1.02 (0.85, 1.22)	1.02 (0.85, 1.22)
Middle(400–800)	2	1892	31.9	0.71 (0.58, 0.87)	0.71 (0.56, 0.91)
Little(<400)	7	3552	50.3	0.72 (0.61, 0.84)	0.69 (0.55, 0.87)
Control source					
Hospital	0				
Population	13	18442	51.2	0.80 (0.72, 0.89)	0.79 (0.68, 0.93)
HWE status					
Meeting HWE	11	16550	56.3	0.79 (0.71, 0.88)	0.81 (0.67, 0.98)
Deviating HWE	2	1892	31.9	0.75 (0.69, 0.82)	0.71 (0.68, 0.91)

### Meta-regression analysis

In order to systematically assess the heterogeneity among the studies, a regression analysis was performed by using the univariate model for the potential sources of heterogeneity including sample size, ethnicity, source of controls, cases definition, mean age of cases, HWE status, genotyping method (RFLP and other methods) and type of study. For the −174G>C genotype, univariate regression analysis showed that HWE status (β coefficient = −0.0169, P = 0.081, I^2^-residual = 28.26%, adj R^2^ = 62.12%) was a significant source of heterogeneity among studies (P<0.1). The other variables were not significant sources of heterogeneity, and all P values exceed 0.1 (Table S5). For the −572G>C genotype, univariate regression analysis showed that sample size (β coefficient = 0.1862, P = 0.04, I^2^-residual = 40.1%, adj-R^2^ = 41.8%) and ethnicity (β coefficient = −0.374, P = 0.02, I^2^-residual = 31.6%, adj-R^2^ = 60.8%) were significant sources of heterogeneity among studies. The other variables were not significant sources of heterogeneity (Table S6).

In addition, we considered an adjusted meta-analysis to evaluate the potential source of heterogeneity from: sample size, ethnicity, type of study and HWE. For the −174G>C polymorphism, after adjusted for sample size, ethnicity, type of study, and HWE, respectively, the pooled results did not change considerably compared to the unadjusted result. The pooled OR (95%CI) value was 0.69 (0.51 to 0.93) for the −572G>C polymorphism after adjustment for ethnicity, and the association between the −572G>C polymorphism and CHD risk was not statistically significant after adjustment for other characteristics (Table S7).

### Publication bias

**Figure 2:**
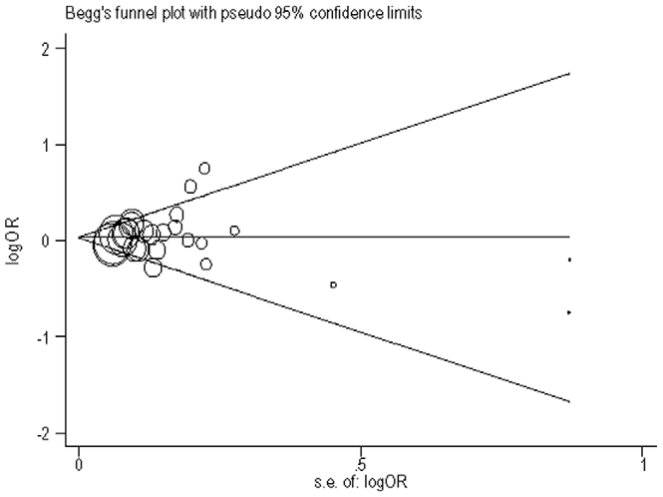
Begg's funnel plot with pseudo 95% confidence limit under the additive genetic model of −174G>C genotype. The size of the circle is proportional to the weight of the study.

**Figure 3:**
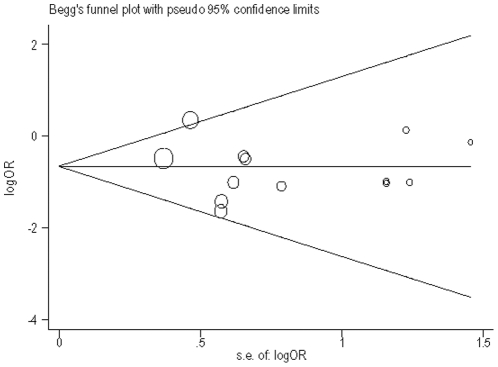
Begg's funnel plot with pseudo 95% confidence limit under the additive genetic model of −572G>C genotype. The size of the circle is proportional to the weight of the study.

We used Egger's method to access the publication bias of the −174G>C and −572G>C polymorphisms in the IL-6 gene and CHD risk under the additive model. Figure 2 showed a funnel plot in which the log value of the OR of CHD risk was plotted against the standard error of the log of the OR in each study on the IL-6 −174G>C polymorphism and CHD. The funnel plot for the overall results was substantially asymmetric for small studies. Moreover, the Egger's test for potential publication bias suggested that there was no significant bias (*t* = 0.5, *P* = 0.619). Figure 3 showed the funnel plot of publication bias on the −572G>C polymorphism and CHD. The plot as a whole is symmetric except for the small studies. The result of Egger's test showed that the publication bias was not obvious (t = −1.29, *P* = 0.215).

## Discussion

Atherosclerosis and CHD may be inflammatory conditions and IL-6 plays a key role in the inflammation process. A meta-analysis of prospective studies showed that the IL-6 plasma levels may be associated with CHD risk [19]. Several studies have also found that the −174C and the −572C alleles are associated with higher serum IL-6 concentrations [30], [70]. One promising hypothesis is that the −174G>C or −572G>C variants of the IL-6 gene are associated with CHD susceptibility. However, results from previous studies on the −174G>C polymorphism and CHD risk were inconsistent. Some studies, such as Bennet AM [32], Basso F [40], Lieb W [47], Nauck M [50], etc, did not find the association between the polymorphism and risk of CHD. Other studies, such as Jenny NS [36], Humphries SE [42], Licastro F [48], Georges JL [51], etc, found a higher risk of CHD associated with the C-allele. However, most of these studies were performed in Western countries on western populations. In addition, different characteristics among studies, such as ethnicity, disease diagnosis criteria, sources of controls, sample size, can introduce heterogeneity, making it hard to interprete the results of association studies. It would thus be difficult to find the robust correlation between the polymorphisms of the IL-6 gene and CHD. In order to find out the origin of heterogeneity and to provide a better overview on the finding of all previous studies, a meta-analysis was performed to analyze a very large number of events.

In the meta analysis of the −174G>C polymorphism, which involved 27812 subjects and 22 studies with 28 comparisons, significant association with CHD risk can not be found in overall comparisons under all genetic models. Results from studies with small sample size or deviating HWE are inconsistent. One explanation may be that small sample size and deviating HWE may cause results bias. Study characteristics, such as means age of cases, genotyping method, study design, source of controls and ethnicity, showed some differences in the included studies. But most of them were not responsible for heterogeneity by subgroup analysis, sensitivity analysis or meta-regression analysis.

Another variant of the IL-6 gene, the −572G>C polymorphism, was recently reported [27] to influence IL-6 gene transcription, thus modulating plasma or tissue levels of IL-6. But no association between this polymorphism and CHD risk was observed in most studies based on Western population [40]–[41], [51]–[52]. It is possible that the frequency of the −572C allele in Western populations is very low. The frequency of the −572C allele was reported to be 0.0055 in Englishman and 0.0019 in Scotland [40], [52], as compared with a frequency of 0.15 in Indian [65] and 0.419 in China [61]. It is also possible that the small samples do not give adequate power to detect the significant risk in carriers. However, the association was significant in Asian population [54]–[58] and the G allele was associated with increased risk for CHD. Our meta-analysis of the −572G>C polymorphism and risk of CHD, which involved in 9498 subjects and 13 studies, showed that the G allele was associated with increased risk for CHD when compared with the C allele under additive recessive model and the CC versus GG genetic model. In the subgroup analysis, associations of the G allele with risk of CHD were not found in studies focusing on European populations, cohort study and studies with large sample size. The heterogeneity among studies was also evident that necessitates further studies.

Overall, a modest association existed between the 572G>C variant and CHD risk. But this association became non-significant when the meta-analysis was restricted to studies on European populations, studies with larger size and cohort studies. In addition, our meta-analysis does not support an association between the 174G>C polymorphism and CHD risk. Compared with a previous meta-analysis [43], the present study is much larger including almost twice as many cases as the earlier meta-analysis. Furthermore, not only the association between the −174G>C polymorphism and CHD risk was assessed, but the association between the −572G>C polymorphism and CHD was also evaluated. Sources of heterogeneity across studies and the possibility of publication bias were systematically explored by using subgroup analysis, sensitivity analysis, meta-regression and Eggers test, etc. Our results indicate an overestimation of the true genetic association by small studies.

Several potential limitations of this meta-analysis should be considered. First, this meta-analysis only focused on papers published in English and Chinese. Second, not all the control subjects were age and sex matched to cases, which was likely one of the causes for heterogeneity. Third, the meta-regression analysis revealed that ethnicity and sample size were the significant sources of heterogeneity across studies on the 572G>C polymorphism and CHD risk. Moreover, because the included studies are observational, confounding and biases might have affected the pooled estimates. However, the advantages of this meta-analysis were also obvious. This study was based on a large sample size of 27 included studies with 11580 cases and 17103 controls. This is twice the sample size that had been reported in the previous meta-analysis. Second, the potential sources of heterogeneity in the meta-analysis were assessed in detail. Third, the associations of two variants of the IL-6 gene with risk of CHD evaluated under different genetic models were similar.

In conclusion, the meta-analysis provides evidence that the −174G>C polymorphism in the IL-6 gene is not significantly associated with increased risk of CHD. A significant association can be found between the −572G>C polymorphism in the IL-6 gene and CHD risk, especially in Asian populations. Furthermore, well-designed large population studies are needed to investigate the relation of these polymorphisms and CHD.

## Supporting Information

Figure S1 **Association between IL-6 gene −174G>C polymorphism and CHD risk under the additive genetic model (tiff).**
(TIF)

Figure S2 **The cumulate meta-analysis between IL-6 gene −174G>C polymorphism and CHD risk under the additive genetic model according to publication year (tiff).**
(TIF)

Figure S3 **Association between IL-6 gene −572G>C polymorphism and CHD risk under the additive genetic model (tiff).**
(TIF)

Table S1 **Basic characteristics of the included studies in the meta-analysis (DOC).**
(DOC)

Table S2 **Detailed characteristics of the included studies in the meta-analysis (DOC).**
(DOC)

Table S3 **The distribution of IL-6 gene −174G>C genotypes and alleles among cases and controls, and P-value of HWE in control (DOC).**
(DOC)

Table S4 **The distribution of IL-6 gene −572G>C genotypes and alleles among cases and control, and P-value of HWE in controls (DOC).**
(DOC)

Table S5 **The meta-regression analysis for heterogeneity under the additive model of IL-6 gene −174G>C polymorphism (DOC).**
(DOC)

Table S6 **The meta-regression analysis for heterogeneity under the additive model of IL-6 gene −572G>C polymorphism (DOC).**
(DOC)

Table S7 **ORs and 95%CI with or without adjusted factors for coronary heart disease and the −174G>C, −572G>C polymorphism in IL-6 gene under additive model (DOC).**
(DOC)
