# Supplementary figures and images for: Polymorphisms of −174G>C and −572G>C in the Interleukin 6 (IL-6) Gene and Coronary Heart Disease Risk: A Meta-Analysis of 27 Research Studies
Source: PLoS One. 2012 Apr 11;7(4):e34839. doi: 10.1371/journal.pone.0034839 (PMC3324545; doi:10.1371/journal.pone.0034839)

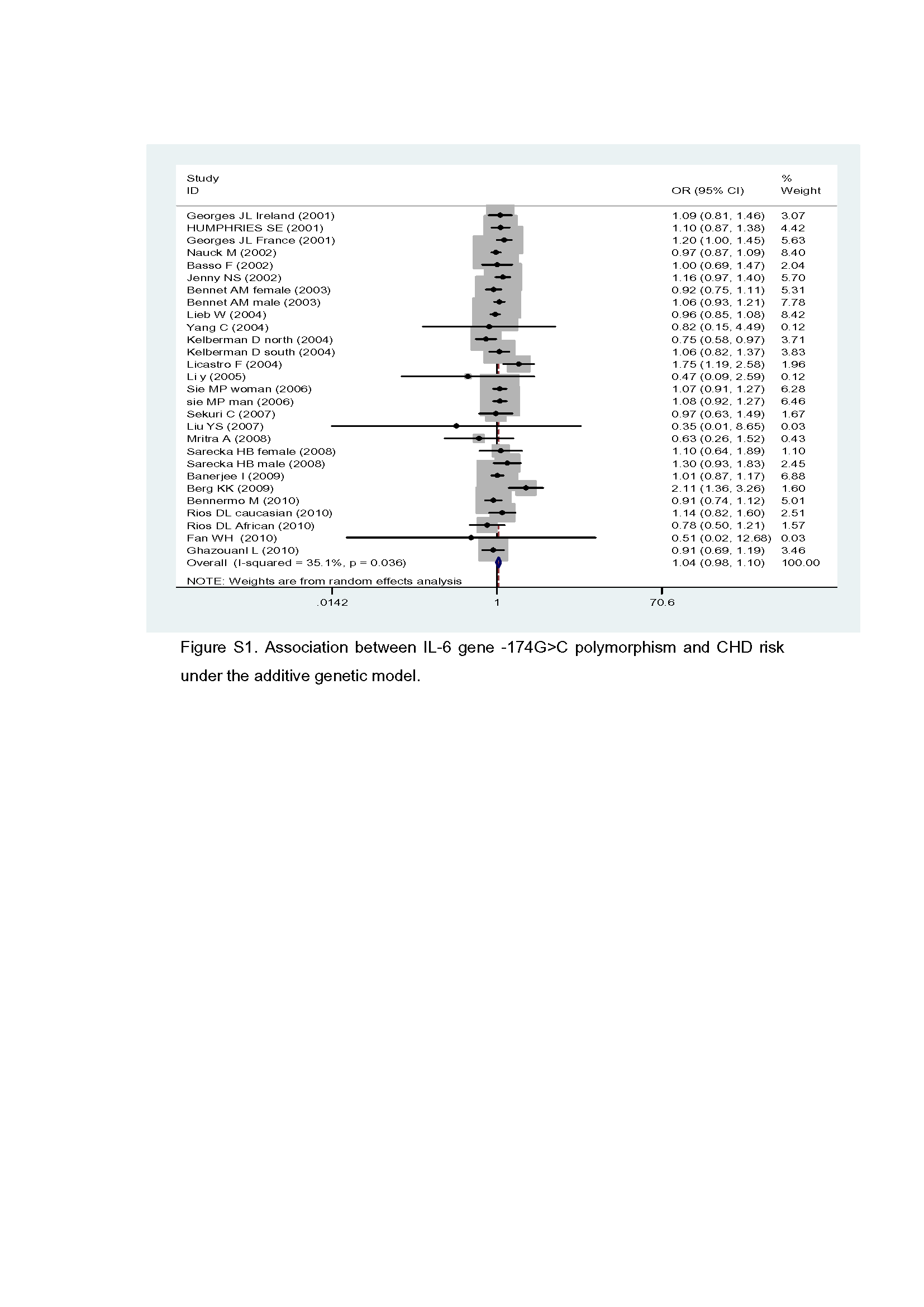

Supplement: Figure S1 — Association between IL-6 gene −174G>C polymorphism and CHD risk under the additive genetic model (tiff). (TIF) [file pone.0034839.s001.tif]

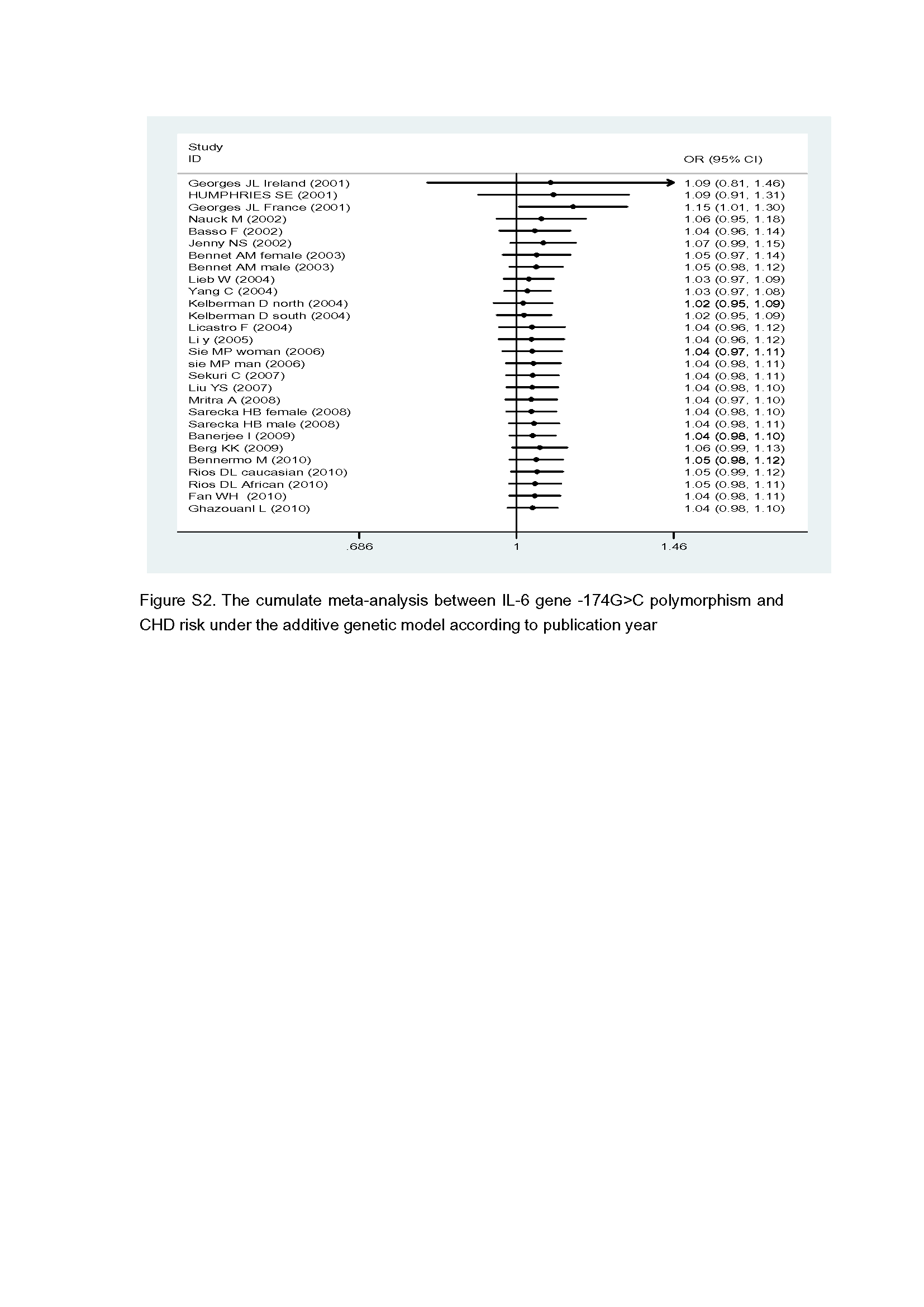

Supplement: Figure S2 — The cumulate meta-analysis between IL-6 gene −174G>C polymorphism and CHD risk under the additive genetic model according to publication year (tiff). (TIF) [file pone.0034839.s002.tif]

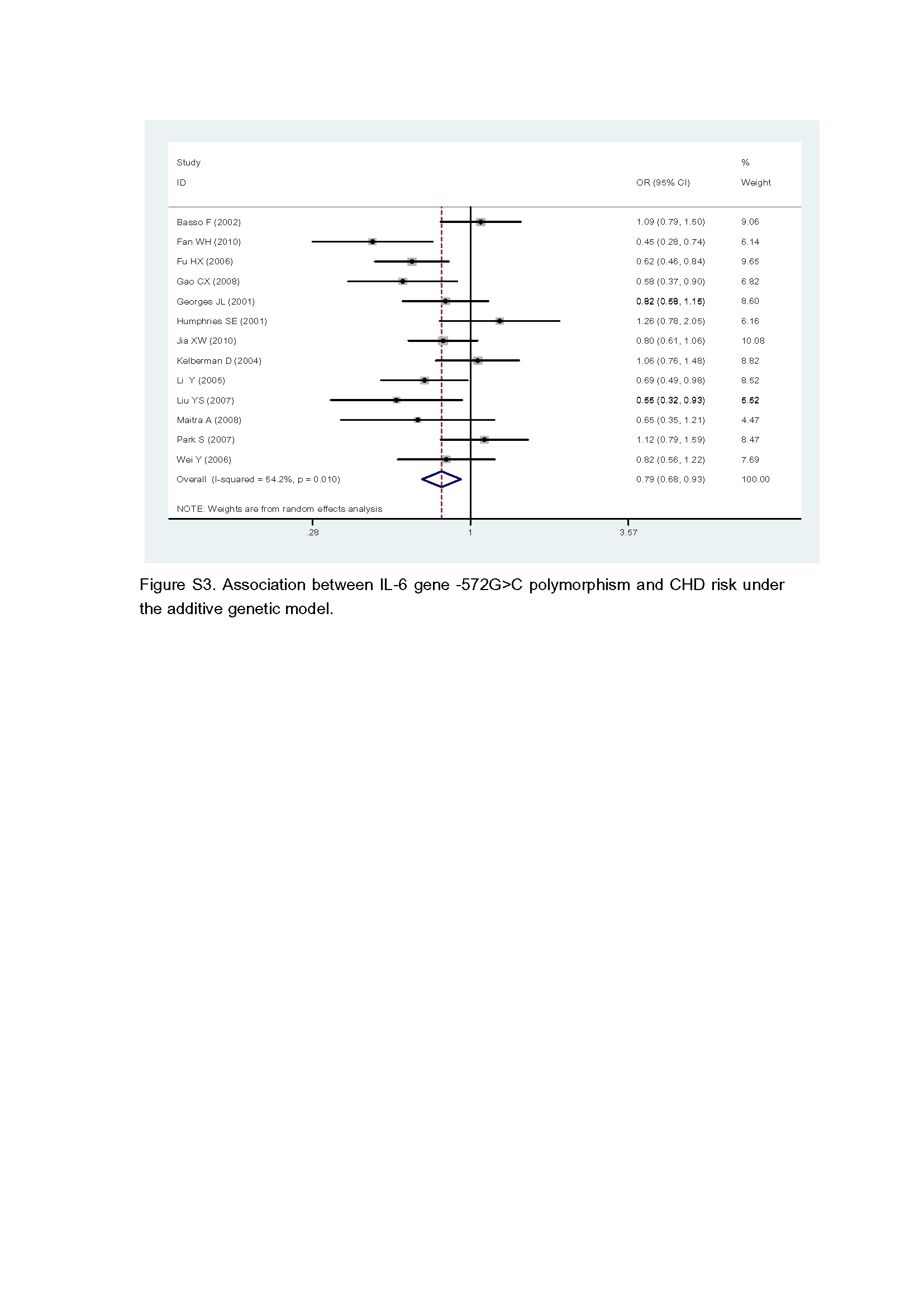

Supplement: Figure S3 — Association between IL-6 gene −572G>C polymorphism and CHD risk under the additive genetic model (tiff). (TIF) [file pone.0034839.s003.tif]
