# Supplementary material for: Polymorphisms of −174G>C and −572G>C in the Interleukin 6 (IL-6) Gene and Coronary Heart Disease Risk: A Meta-Analysis of 27 Research Studies
Source: PLoS One. 2012 Apr 11;7(4):e34839. doi: 10.1371/journal.pone.0034839 (PMC3324545; doi:10.1371/journal.pone.0034839)
Supplement: Table S1 — Basic characteristics of the included studies in the meta-analysis (DOC). (DOC) [file pone.0034839.s004.doc]

| Table S1. Basic characteristics of the included studies in the meta-analysis | | | | | | | | |
| --- | --- | --- | --- | --- | --- | --- | --- | --- |
| Study included | year | Country | Ethnicity | Gender component M/F | | Age(Mean±SD),Year | | |
|  |  |  |  | cases | controls | cases | controls | |
| Basso F^[40]^ | 2002 | Scotland | European | M | M | 56±5 | | 56±5 |
| Jenny NS^[41]^ | 2002 | America | American | 215/250 | 193/298 | 73.3 | | 72.3 |
| Humphries SE^[42]^ | 2001 | England | European | No clear | No clear | 56.7±3.6 | | 56.0±3.4 |
| Sie MP^[43]^ | 2006 | Netherlands | European | No clear | No clear | No clear | | No clear |
| Berg KK^[44]^ | 2009 | Norwegian | European | 106/24 | 36/64 | 60 | | 57 |
| Sekuri C^[33]^ | 2007 | Turk | European | 88/27 | 83/22 | 46.3±7.8 | | 44.3±7.2 |
| Rios DL^[45]^ | 2010 | Brazil | African | 89/48 | 50/65 | 55.7±7.9 | | 51.8±8.4 |
|  |  |  | European | 97/179 | 26/112 | 55.7±6.7 | | 53.0±7.7 |
| Banerjee I^[46]^ | 2009 | India | Asian | 166/44 | 166/66 | 56.3±12.1 | | 56.0±9.5 |
| Lieb W^[47]^ | 2004 | Germany | European | 986/336 | 471/552 | 57±8 | | 52±13 |
| Licastro F^[48]^ | 2004 | Italia | European | No clear | No clear | 65±11 | | 57±9 |
| Bennet AM^[32]^ | 2003 | Swedish | European | 852/361 | 1054/507 | No clear | | No clear |
| Man |  |  |  | 852 | 1054 | 59 | | 59 |
| Woman |  |  |  | 361 | 507 | 63 | | 63 |
| Ghazouani L^[49]^ | 2010 | Tunis | European | 331/87 | 299/107 | 58.1±12.0 | | 56.7±14.1 |
| Nauck M^[50]^ | 2002 | Germany | European | 1928/653 | 1091/296 | 63.8±9.89 | | 58.3±11.83 |
| Georges JL^[51]^ | 2001 | England | European |  |  |  | |  |
| Ireland |  |  |  | No clear | No clear | 54.4±7.9 | | 54.1±7.8 |
| France |  |  |  | No clear | No clear | 54.0±8.3 | | 53.3±8.6 |
| Kelberman D^[52]^ | 2004 | England | European |  |  |  | |  |
| North |  |  |  | No clear | No clear | 53.1±5.1 | | 52.7±5.0 |
| South |  |  |  | No clear | No clear | 50.9±5.5 | | 50.5±5.6 |
| Li Y^[53]^ | 2005 | China | Asian | 127/72 | 110/79 | 56±17 | | 53±19 |
| Wei YS^[54]^ | 2006 | China | Asian | 125/40 | 127/43 | 61±11 | | 60±10 |
| Fu HX^[55]^ | 2006 | China | Asian | 170/75 | 181/79 | 61.8±12.4 | | 59.89±14.4 |
| Liu YS^[58]^ | 2007 | China | Asian | 58/32 | 54/41 | 65.59±9.1 | | 57.02±6.98 |
| Yang C^[59]^ | 2004 | China | Asian | 69/43 | 106/77 | 55±14 | | 52±18 |
| Gao CX^[63]^ | 2008 | China | Asian | 79/47 | 75/33 | 65.2±9.8 | | 62.5±11.8 |
| Jia XW^[64]^ | 2010 | China | Asian | 156/75 | 95/115 | 51.4±10.6 | | 49.5±10.01 |
| Maitra A^[65]^ | 2008 | India | Asian | 239/45 | 31/9 | 57.17±9.2 | | 45.53±5.98 |
| Park S^[66]^ | 2007 | Korean | Asian | 129/41 | 114/56 | 62.2±11.4 | | 62.6±10.4 |
| Sarecka HB^[67]^ | 2008 | Caucasian | European | 119/58 | 154/48 | 43.8±6.1 | | 35.4±10.4 |
| Fan WH^[68]^ | 2010 | China | Asian | 45/39 | 76/54 | 52.1±6.8 | | 52.3±8.8 |
| Bennermo M^[69]^ | 2010 | Switzerland | European | 298/66 | 298/66 | 54(49~57) | | 54(49~57) |
