# Supplementary material for: Polymorphisms of −174G>C and −572G>C in the Interleukin 6 (IL-6) Gene and Coronary Heart Disease Risk: A Meta-Analysis of 27 Research Studies
Source: PLoS One. 2012 Apr 11;7(4):e34839. doi: 10.1371/journal.pone.0034839 (PMC3324545; doi:10.1371/journal.pone.0034839)
Supplement: Table S2 — Detailed characteristics of the included studies in the meta-analysis (DOC). (DOC) [file pone.0034839.s005.doc]

| Table S2. Detailed characteristics of the included studies in the meta-analysis | | | | | |
| --- | --- | --- | --- | --- | --- |
| Study included | Type of study | Genotyping method | Sample  size | Control source | End point of assessment |
| Basso F | Case control | SSCP | Large | Population | MI for WHO criteria; CHD for stenosis ≥50% |
| Jenny NS | Case control | RFLP | Large | Population | CAD with MRI-detectable infarcts |
| Humphries SE | Cohort study | SSCP | Large | Population | CHD and MI based on WHO criteria |
| Sie MP | Cohort study | RFLP | Large | Population | CHD and MI based on international classification of disease, 10^th^ edition. |
| Berg KK | Case control | RFLP | Little | Hospital | CHD for coronary artery stenosis ≥50% |
| Sekuri C | Case control | RFLP | Little | Hospital | CAD was defined as stenosis ≥50% and MI for WHO criteria |
| Rios DL | Case control | RFLP | Middle | Hospital | CAD at least one obstructive lesion ≥50% |
| Banerjee I | Case control | RFLP | Middle | Population | CHD for stenosis ≥50% |
| Lieb W | Case control | RFLP | large | Population | MI defined by the MONICA diagnosis criteria |
| Licastro F | Case control | RFLP | little | Population | MI for WHO criteria |
| Bennet AM | Case control | DASH | large | Population | MI for WHO criteria |
| Ghazouani L | Case control | RFLP | large | Population | CAD for at least stenosis ≥50% and MI for WHO criteria |
| Nauck M | Case control | RFLP | large | Population | CAD was defined by using angiographic criteria (stenosis ≥50%)and ECG criteria |
| Georges JL | Case control | RFLP | large | Population | MI for MONICA criteria and CAD for ≥50% stenosis |
| Kelberman D | Case control | RFLP | large | Population | MI for WHO criteria |
| Li Y | Case control | RFLP | little | Population | CHD for WHO criteria |
| Wei YS | Case control | RFLP | little | Population | CHD for WHO criteria |
| Fu HX | Case control | RFLP | Middle | Population | CHD with stenosis≥50% |
| Liu YS | Case control | RFLP | little | Population | CHD with stenosis ≥50% |
| Yang C | Case control | RFLP | little | Population | CHD with WHO criteria |
| Gao CX | Case control | RFLP | little | Population | CHD with WHO criteria or stenosis ≥50% |
| Jia XW | Case control | RFLP | Middle | Population | CHD was diagnosed by coronary arteriography (stenosis ≥50%) |
| Maitra A | Case control | Real- time | little | Population | CAD for at least stenosis ≥50% and MI for WHO criteria |
| Park S | Case control | RFLP | little | Population | AMI with WHO critera |
| Sarecka HB | Case control | RFLP | little | Population | CAD with stenosis ≥50% |
| Fan WH | Case control | RFLP | little | Population | CHD with stenosis≥50% |
| Bennermo M | Case control | TaqMan | Middle | Population | CHD with WHO criteria |
| MI: myocardial infarction; CAD: Coronary artery disease; CHD: Coronary heart disease; MONICA: Multinational Monitoring of Trends and Determinants in Cardiovascular Disease Project; AMI: Acute myocardial infarction | | | | | |
