# Supplementary material for: Polymorphisms of −174G>C and −572G>C in the Interleukin 6 (IL-6) Gene and Coronary Heart Disease Risk: A Meta-Analysis of 27 Research Studies
Source: PLoS One. 2012 Apr 11;7(4):e34839. doi: 10.1371/journal.pone.0034839 (PMC3324545; doi:10.1371/journal.pone.0034839)
Supplement: Table S3 — The distribution of IL-6 gene −174G>C genotypes and alleles among cases and controls, and P-value of HWE in control (DOC). (DOC) [file pone.0034839.s006.doc]

| Table S3. The distribution of IL-6 gene -174G>C genotypes and alleles among cases and controls, and P-value of HWE in control | | | | | | | | | | | | |
| --- | --- | --- | --- | --- | --- | --- | --- | --- | --- | --- | --- | --- |
|  |  |  |  |  |  |  |  |  |  | C allele | | HWE |
| Studies | Numbers | | | Case genotype | | | Control genotype | | | frequency % | | *P* value |
| included | Cases | Controls | Total | GG | GC | CC | GG | GC | CC | Cases | Controls | Controls |
| Basso F | 498 | 1107 | 1607 | 161 | 259 | 78 | 375 | 549 | 185 | 15.66 | 16.71 | 0.505 |
| Jenny NS | 465 | 491 | 956 |  |  | 194 |  |  | 187 | 41.72 | 38.09 | No calculated |
| Humphries SE | 162 | 2589 | 2751 | 40 | 95 | 25 | 827 | 1263 | 470 | 15.63 | 18.36 | 0.75 |
| Sie MP | 671 | 5013 | 5630 | 231 | 319 | 121 | 1815 | 2354 | 844 | 18.03 | 16.84 | 0.10 |
| Man | 362 | 1810 | 2172 | 128 | 170 | 64 | 673 | 849 | 288 | 17.68 | 15.91 | 0.459 |
| woman | 309 | 3203 | 3458 | 103 | 149 | 57 | 1142 | 1505 | 556 | 18.45 | 17.36 | 0.117 |
| Berg KK | 130 | 100 | 230 | 87 | | 43 | 81 | | 19 | 33.08 | 19.00 | No calculated |
| Sekuri C | 115 | 105 | 220 | 61 | 49 | 5 | 57 | 41 | 7 | 4.35 | 6.67 | 0.919 |
| Rios DL | 414 | 253 | 657 | 254 | 126 | 34 | 151 | 89 | 13 | 8.21 | 5.14 | 0.980 |
| African-B | 138 | 115 | 243 | 96 | 36 | 6 | 69 | 43 | 3 | 4.35 | 2.61 | 0.217 |
| Caucasian-B | 276 | 138 | 414 | 158 | 90 | 28 | 82 | 46 | 10 | 10.14 | 7.25 | 0.324 |
| Banerjee I | 210 | 232 | 442 | 159 | 43 | 8 | 171 | 57 | 4 | 3.81 | 1.72 | 0.763 |
| Lieb W | 1322 | 1023 | 2345 | 451 | 627 | 244 | 331 | 499 | 193 | 18.46 | 18.87 | 0.839 |
| Licastro F | 139 | 198 | 337 | 35 | 88 | 15 | 46 | 44 | 7 | 10.87 | 7.22 | 0.418 |
| Bennet AM | 1213 | 1561 | 2774 | 305 | 577 | 275 | 398 | 754 | 348 | 23.77 | 23.20 | 0.803 |
| Man | 852 | 1054 | 1825 | 210 | 402 | 200 | 278 | 500 | 235 | 24.63 | 23.20 | 0.725 |
| Woman | 361 | 507 | 832 | 95 | 175 | 75 | 120 | 254 | 113 | 21.74 | 23.20 | 0.339 |
| Ghazouani L | 418 | 406 | 824 | 297 | 102 | 7 | 298 | 110 | 10 | 1.72 | 2.39 | 0.968 |
| Nauck M | 2575 | 729 | 3304 | 838 | 1238 | 499 | 230 | 355 | 144 | 19.38 | 19.75 | 0.739 |
| Georges JL | 414 | 612 | 1026 | 170 | 340 | 104 | 231 | 336 | 105 | 16.94 | 15.63 | 0.345 |
| Ireland | 190 | 180 | 370 | 45 | 109 | 32 | 47 | 97 | 28 | 17.20 | 16.28 | 0.063 |
| France | 224 | 432 | 656 | 125 | 231 | 72 | 184 | 239 | 77 | 16.82 | 15.40 | 0.966 |
| Kelberman D | 587 | 562 | 1079 | 227 | 219 | 61 | 240 | 240 | 81 | 12.03 | 14.44 | 0.10 |
| North | 229 | 244 | 473 | 89 | 100 | 40 | 71 | 120 | 53 | 17.47 | 21.72 | 0.863 |
| South | 288 | 318 | 606 | 138 | 119 | 21 | 169 | 120 | 28 | 7.55 | 8.83 | 0.317 |
| Li Y | 199 | 189 | 388 | 197 | 2 | 0 | 185 | 4 | 0 | 0.00 | 0.00 | 0.883 |
| Liu YS | 90 | 95 | 185 | 90 | 0 | 0 | 94 | 1 | 0 | 0.00 | 0.00 | 0.959 |
| Yang C | 112 | 183 | 295 | 110 | 2 | 0 | 179 | 4 | 0 | 0.00 | 0.00 | 0.881 |
| Maitra A | 284 | 40 | 324 | 36 | 10 | 0 | 30 | 7 | 3 | 0.00 | 7.50 | 0.024 |
| Sarecka HB | 177 | 202 | 379 | 43 | 92 | 42 | 60 | 105 | 37 | 23.73 | 18.32 | 0.449 |
| Man | 119 | 154 | 273 | 27 | 62 | 30 | 50 | 78 | 26 | 25.21 | 16.88 | 0.635 |
| Woman | 58 | 48 | 106 | 16 | 30 | 12 | 10 | 27 | 11 | 20.69 | 22.92 | 0.385 |
| Fan WH | 84 | 130 | 210 | 84 | 0 | 0 | 129 | 1 | 0 | 0.00 | 0.00 | 0.965 |
| Bennermo M | 364 | 364 | 728 | 119 | 150 | 87 | 109 | 176 | 93 | 24.44 | 24.60 | 0.192 |
