# Supplementary material for: Polymorphisms of −174G>C and −572G>C in the Interleukin 6 (IL-6) Gene and Coronary Heart Disease Risk: A Meta-Analysis of 27 Research Studies
Source: PLoS One. 2012 Apr 11;7(4):e34839. doi: 10.1371/journal.pone.0034839 (PMC3324545; doi:10.1371/journal.pone.0034839)
Supplement: Table S4 — The distribution of IL-6 gene −572G>C genotypes and alleles among cases and control, and P-value of HWE in controls (DOC). (DOC) [file pone.0034839.s007.doc]

| Table S4. The distribution of IL-6 gene -572G>C genotypes and alleles among cases and control, and P-value of HWE in controls | | | | | | | | | | | | |
| --- | --- | --- | --- | --- | --- | --- | --- | --- | --- | --- | --- | --- |
|  |  |  |  |  |  |  |  |  |  | C allele | | HWE |
|  | Numbers | | | Case genotype | | | Control genotype | | | frequency % | | *P* value |
| First author | Cases | Controls | Total | GG | GC | CC | GG | GC | CC | Cases | Controls | Controls |
| Basso F | 498 | 1108 | 1606 | 425 | 56 | 1 | 959 | 116 | 2 | 0.21 | 0.19 | 0.437 |
| Humphries SE | 162 | 2589 | 2751 | 135 | 19 | 0 | 2224 | 225 | 9 | 0.00 | 0.37 | 0.199 |
| Georges JL | 414 | 612 | 1026 | 552 | 58 | 1 | 589 | 73 | 3 | 0.16 | 0.45 | 0.650 |
| Kelberman D | 587 | 562 | 1079 | 433 | 71 | 1 | 475 | 69 | 3 | 0.20 | 0.55 | 0.774 |
| Li Y | 199 | 189 | 388 | 16 | 64 | 119 | 4 | 60 | 125 | 59.80 | 66.14 | 0.297 |
| Wei YS | 165 | 170 | 235 | 6 | 54 | 105 | 4 | 50 | 116 | 63.64 | 68.24 | 0.608 |
| Fu HX | 245 | 260 | 505 | 16 | 101 | 128 | 4 | 90 | 166 | 52.24 | 63.85 | 0.034 |
| Liu YS | 90 | 95 | 185 | 2 | 39 | 49 | 1 | 26 | 68 | 54.44 | 71.58 | 0.385 |
| Gao CX | 126 | 108 | 234 | 10 | 51 | 65 | 4 | 32 | 72 | 51.59 | 66.67 | 0.85 |
| Jia XW | 231 | 210 | 441 | 22 | 130 | 79 | 15 | 107 | 88 | 34.20 | 41.90 | 0.021 |
| Maitra A | 284 | 40 | 324 | 23 | 15 | 8 | 11 | 23 | 6 | 17.39 | 15.00 | 0.287 |
| Park S | 170 | 170 | 340 | 9 | 62 | 97 | 12 | 62 | 92 | 57.74 | 55.42 | 0.728 |
| Fan WH | 84 | 130 | 210 | 4 | 38 | 42 | 3 | 32 | 95 | 50.00 | 73.08 | 0.875 |
