# Supplementary material for: Polymorphisms of −174G>C and −572G>C in the Interleukin 6 (IL-6) Gene and Coronary Heart Disease Risk: A Meta-Analysis of 27 Research Studies
Source: PLoS One. 2012 Apr 11;7(4):e34839. doi: 10.1371/journal.pone.0034839 (PMC3324545; doi:10.1371/journal.pone.0034839)
Supplement: Table S5 — The meta-regression analysis for heterogeneity under the additive model of IL-6 gene −174G>C polymorphism (DOC). (DOC) [file pone.0034839.s008.doc]

| Table S5. The meta-regression analysis for heterogeneity under the additive model of IL-6 gene -174G>C polymorphism | | | | | | |
| --- | --- | --- | --- | --- | --- | --- |
| Variable | Coefficient | T value | P value | Tau2 value | I^2^%  (residual) | Adj R^2^  % |
| Sample size | -0.042 | -0.96 | 0.346 | 0.0045 | 34.6 | -18.9 |
| Ethnicity | 0.013 | 0.25 | 0.802 | 0.0045 | 35.9 | -18.6 |
| Type of study | -0.049 | -0.61 | 0.550 | 0.0042 | 35.0 | -11.3 |
| Genotyping method | 0.045 | 0.64 | 0.531 | 0.0062 | 36.3 | -63.2 |
| Cases  definition | -0.014 | -0.34 | 0.736 | 0.0057 | 36.5 | -51.7 |
| Mean age  Of cases | 0.004 | 0.59 | 0.558 | 0.0096 | 39.9 | -27.2 |
| Sources of control | -0.119 | -0.95 | 0.349 | 0.0034 | 34.3 | 8.9 |
| HWE-status | -0.169 | -1.81 | 0.081 | 0.00143 | 28.26 | 62.12 |
| Tau2: estimate of between-study variance; I^2^ % (residual): % residual variation due to heterogeneity; Adj-R^2^: proportion of between-study variance explained | | | | | | |
