# Supplementary material for: Polymorphisms of −174G>C and −572G>C in the Interleukin 6 (IL-6) Gene and Coronary Heart Disease Risk: A Meta-Analysis of 27 Research Studies
Source: PLoS One. 2012 Apr 11;7(4):e34839. doi: 10.1371/journal.pone.0034839 (PMC3324545; doi:10.1371/journal.pone.0034839)
Supplement: Table S6 — The meta-regression analysis for heterogeneity under the additive model of IL-6 gene −572G>C polymorphism (DOC). (DOC) [file pone.0034839.s009.doc]

| Table S6. The meta-regression analysis for heterogeneity under the additive model of IL-6 gene -572G>C polymorphism | | | | | | |
| --- | --- | --- | --- | --- | --- | --- |
| Variable | Coefficient | T value | P value | Tau2 value | I^2^%  (residual) | Adj R^2^  % |
| Sample size | 0.1862 | 2.32 | 0.040 | 0.0258 | 40.1 | 41.8 |
| Ethnicity | -0.3740 | -2.67 | 0.021 | 0.0172 | 31.6 | 60.8 |
| Type of study | -0.4966 | -1.51 | 0.158 | 0.0385 | 51.5 | 13.2 |
| Genotyping method | -0.2121 | -0.78 | 0.452 | 0.0459 | 56.1 | -3.5 |
| Cases  definition | 0.1119 | 1.25 | 0.237 | 0.0362 | 49.8 | 18.5 |
| Mean age  of cases | -0.0145 | -0.73 | 0.487 | 0.0472 | 55.5 | -6.3 |
| HWE-status | 0.1369 | 0.64 | 0.537 | 0.0486 | 54.86 | -9.7 |
| Tau2: estimate of between-study variance; I^2^ % (residual): % residual variation due to heterogeneity; Adj-R^2^: proportion of between-study variance explained | | | | | | |
