# Supplementary material for: Polymorphisms of −174G>C and −572G>C in the Interleukin 6 (IL-6) Gene and Coronary Heart Disease Risk: A Meta-Analysis of 27 Research Studies
Source: PLoS One. 2012 Apr 11;7(4):e34839. doi: 10.1371/journal.pone.0034839 (PMC3324545; doi:10.1371/journal.pone.0034839)
Supplement: Table S7 — ORs and 95%CI with or without adjusted factors for coronary heart disease and the −174G>C, −572G>C polymorphism in IL-6 gene under additive model (DOC). (DOC) [file pone.0034839.s010.doc]

| Table S7. ORs and 95%CI with or without adjusted factors for coronary heart disease and the -174G>C, -572G>C polymorphism in IL-6 gene under additive model | | | | | |
| --- | --- | --- | --- | --- | --- |
| Adjusted Variables | -174G>C polymorphism | |  | -572G>C polymorphism | |
|  | OR values | 95%CI |  | OR values | 95%CI |
| Unadjusted | 1.04 | 0.98~1.10 |  | 0.79 | 0.68~0.93 |
| Adjusted variables |  |  |  |  |  |
| Sample size | 0.96 | 0.88~1.05 |  | 1.21 | 1.00~1.44 |
| Ethnicity | 1.01 | 0.91~1.12 |  | 0.69 | 0.51~0.93 |
| HWE-status | 0.84 | 0.70~1.02 |  | 1.15 | 0.72~1.84 |
| Type of study | 0.95 | 0.81~1.12 |  | 0.61 | 0.30~1.25 |
| Genotyping method | 1.05 | 0.91~1.21 |  | 0.81 | 0.44~1.47 |
| Mean age of cases | 1.00 | 0.99~1.02 |  | 0.99 | 0.94~1.03 |
